# AWaRe-based culture reporting format: a novel tool for antimicrobial stewardship

**DOI:** 10.3205/id000084

**Published:** 2023-11-14

**Authors:** Heera Hassan, Jyothi R, Sreenadh H, Manjusree S, Aravind Reghukumar

**Affiliations:** 1 Department of Microbiology, Government Medical College, Thiruvananthapuram, Kerala, India; 2 Department of Infectious Diseases, Government Medical College, Thiruvananthapuram, Kerala, India

## Letter to the editor

Dear editor,

We are writing this letter to introduce a novel antimicrobial stewardship tool conceptualized and piloted in the Government Medical College Hospital, Thiruvananthapuram, Kerala State, India. The aim of this tool is to familiarize the prescribing physicians with the World Health Organization’s (WHO) AWaRe classification of antibiotics. We believe that this approach has the potential to significantly optimize the current prescription practices with positive impact on antimicrobial stewardship and thereby patient outcomes in the field of infectious diseases.

In recent years, the rise of antimicrobial resistance has emerged as a significant global concern, challenging the efficacy of our most powerful tools in combating infectious diseases [1]. Antimicrobial stewardship programs have been implemented in healthcare settings to promote the appropriate and responsible use of antibiotics, with the aim of preserving their effectiveness for future generations [2]. However, despite these efforts, the problem of antimicrobial resistance continues to escalate.

In order to support antibiotic stewardship efforts at local, national and global levels, in 2017 the WHO Expert Committee on Selection and Use of Essential Medicines developed the AWaRe classification of antibiotics with the aim of emphasizing the importance of appropriate antibiotic selection [3]. The AWaRe framework is based on a spectrum of activity and resistance potential of antibiotics [4]. The WHO AWaRe tool recommends that by 2023, 60% of antibiotic prescription should be from the Access group [5]. Multiple studies have shown that awareness of the AWaRe tool among prescribers is sub-optimal and hence novel methods for continuous prescriber engagement are needed [6]. 

**Figure 1:**
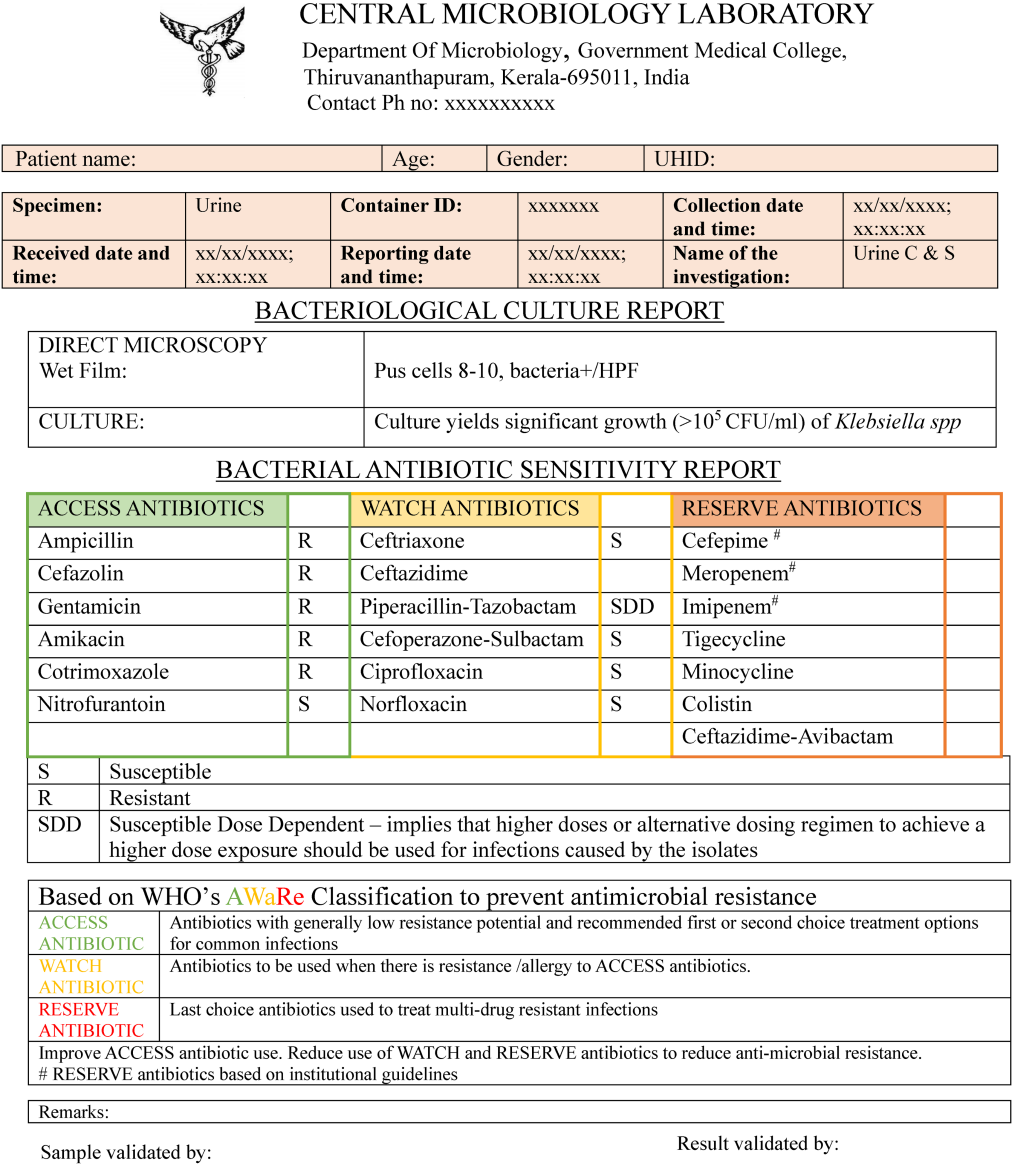
AWaRe-based bacteriological culture report

In light of this pressing issue, we devised a new antibiotic stewardship tool in the form of an AWaRe-based bacterial culture susceptibility reporting format (Figure 1 (Fig. 1)) instead of the conventional format in which antibiotics are sorted into first, second and third line. Clear footnotes have been included with definitions of ACCESS, WATCH, and RESERVE drugs so as to ensure optimization of antimicrobial stewardship by prescribers. In this reporting format, each positive culture report will act as a continuous reminder to the prescribers to select susceptible antibiotics from the Access category. These incessant reminders and the cascade reporting associated with positive culture reports will enhance the knowledge, attitudes, and practices of healthcare professionals and thereby augment antibiotic stewardship practices.

The main advantages of this novel tool include:

1. The AWaRe-based culture reporting format serves as a platform for the dissemination of knowledge regarding optimal antibiotic prescription practices and thereby will help in achieving the WHO target of having 60% of antibiotic prescription from the Access category. Each positive culture report will be like a revision of the AWaRe tool for the prescriber and hence each culture report becomes a stewardship tool.
2. The inclusion of the list of antibiotics in the Access, Watch, and Reserve classes enables healthcare professionals to make quick decisions regarding antibiotic selection while considering antimicrobial stewardship policies and patient safety. This eliminates the need for additional effort to search for the list of antibiotics that need to be preserved for future use, even for doctors who are interested in following antimicrobial stewardship.
3. All healthcare professionals indirectly participate in antimicrobial stewardship programs.
4. It establishes a foundation for antimicrobial prescription audits, allowing healthcare professionals to be questioned about their choices if susceptible Access antibiotics are not prescribed.
5. It helps to dispel misconceptions, such as the belief that vancomycin is superior to penicillin/ampicillin.

We strongly believe that integrating the AWaRe-based culture reporting format into microbiology culture and antibiotic susceptibility reports has the potential to revolutionize antibiotic stewardship practices and might have a significant impact on antimicrobial resistance rates. Developing and adopting such a strategy for report formats while adhering to national/international standards (such as by the Clinical and Laboratory Standards Institute (CLSI) or the European Committee on Antimicrobial Susceptibility Testing (EUCAST)) for interpreting antimicrobial susceptibility testing can be highly beneficial.

## Notes

### Competing interests

The authors declare that they have no competing interests.
